# Art Therapy for Children in Short-Term Hospitalization

**DOI:** 10.5334/cie.63

**Published:** 2023-06-15

**Authors:** Meirav Hen

**Affiliations:** 1 Tel-Hai Academic College, Israel

**Keywords:** Hospitalized children, art therapy, short-term therapy

## Abstract

Pediatric hospitalization is known to be associated with adverse developmental and psychosocial outcomes for young patients. Art therapy is a direct means of addressing the emotional world of the hospitalized child. However, the hospital setting sometimes requires adaptations of this practice to a short-term mode. To explore the characteristics of the adjusted short-term art therapy mode, 10 experienced art therapists who work with hospitalized children were interviewed. Results highlighted the unique aspects of working with children who enter short-term hospitalization, addressing the unknown but most likely brief duration of art therapy and the issues involved, such as the need to use problem-focused strategies, the diffused therapeutic space, interaction with a multidisciplinary staff, and parent’s presence during the therapy session. Integrating the principles of art therapy with the principles of short-term therapy, this paper explores a model for carrying out short-term art therapy with children undergoing short hospitalization.

Many children experience short or prolonged hospitalization during their development (Hen, 2022a). The experience of hospitalization presents the child not only with the pain and anxiety associated with the medical condition, but also with a significant change in the daily routine, distance from family and friends, and loss of school days (Dalei et al., 2020). Hospitalized children report feelings of helplessness, confusion, and tension, as well as the need for a mature and professional figure to mediate the experience of hospitalization and encourage cooperation with medical treatment and recovery processes (Caleffi et al., 2016).

In recognition of the need for hospitalized children to maintain a routine even when they are unable to attend school, educational services have been established in pediatric medical centers in Israel (Hen & Gilan Shochat, 2022b). In these educational services, special education teachers provide support for the child’s learning while art therapists provide the child with emotional support (Weinfeld, 2013).

For many children, art is a natural and spontaneous expression tool that helps them develop proper internal and interpersonal communication (Regev et al., 2015). Aided by creative materials, art therapy allows children to express concerns, emotions and thoughts that are sometimes difficult to express in words (Deboys et al., 2017). Through therapeutic practice and creative materials, the therapist helps children process their experiences and cope with the changes in their daily lives (Clapp et al., 2018).

However, unlike traditional art therapy, which is usually a long-term pre-planned emotional process in a specific setting, art therapy in the hospital educational services takes place as the result of an immediate need, and in whatever setting the hospital can offer and the child can access (Weinfeld, 2013). Educational centers in Israel serve more than 250,000 hospitalized children every year, and many of these admissions range from a few days to two weeks (Hen & Gilan Shochat, 2022b). Therefore, art therapists in the educational services need to adjust the treatment and create a short-term but meaningful therapeutic experience.

The aim of the present study was to explore and better understand the various aspects of a short-term mode of art therapy for hospitalized children. This unique practice was discussed by 10 experienced art therapists who work in the in-hospital educational services, with the aim of contributing to the development of a clear short-term art therapy working model.

## Role and Effect of Art Therapy in Hospital Settings

Art therapy is an emotional support process that utilizes visual and nonverbal symbols for emotional adjustment and transformation (Holmqvist et al., 2017; Wood et al., 2011). The process in this type of therapy mobilizes the language of plastic arts, such as painting, sculpture, or drawing, to achieve personal and developmental change while providing opportunities for expressing emotions (Regev, 2022). The nonverbal work involved in art therapy serves as a means of communication that bypasses verbal language, helping to expose and understand mental processes, as well as provide an environment in which conscious and unconscious issues can surface (Regev et al., 2015).

Studies show that art therapy, especially its creative expression, provides a safe foundation for expression of thoughts and feelings, thus facilitating recovery in times of psychological distress (Metzl, 2022). The creative work serves as a mediating tool between the therapist and the patient, allowing images to speak about the patient’s experience (Chambala, 2008). In addition, it has been found that art itself and being involved in art has therapeutic value based on the trust between therapist and patient that increases personal the well-being and psychological functioning of the patient (Malchiodi, 2008).

In recent years art therapy has become a tool of personal empowerment, in which participants strengthen their understanding of their personal boundaries in relation to the needs of others (Wood et al., 2011). This tool also helps increase the sense of belonging, self-esteem, and self-confidence (Deboys et al., 2017). Art therapy serves as a treatment and intervention and is implemented in the community in institutions such as nursing homes, formal schools, mental health centers, and hospitals (Belity et al., 2017; Shella, 2018).

The presence of expressive therapy programs in hospitals has become widespread in the 21st century (Councill, 2012). Art therapists often work jointly with staff in medical settings, providing interventions that are complementary to traditional medical interventions (Dionigi & Gremigni, 2016). Hospitals increasingly use art therapy to enhance wellness and provide relief not only for the hospitalized child, but also for family members who accompany them (Clapp et al., 2018). Participation in creative work while in a medical framework can act as a diversion and offer emotional support for children, helping them to redevelop their sense of hope, strength, and self-esteem (Regev, 2022). For instance, one study found that the act of drawing increased children’s awareness of their health conditions and their capacity to problem solve and cope with their illnesses (Aguilar, 2017). Similarly, in another study drawing activities were found to enhance communication, understanding of the illness, coping, and children’s emotional expression, and parents reported a reduction in children’s pain levels and nausea in a transfusion room (Madden et al., 2010). Further, Beebe and colleagues (2010) observed that art therapy decreased anxiety for children suffering from asthma compared to a control group, and Metzl et al. (2016) found that both art and music therapy showed some efficacy in altering pain and mood in hospitalized children. Clapp et al. (2018) argued that distracting the hospitalized child from the illness allows a creative process that leads the child to develop coping solutions along with an improved sense of self-worth. The use of creative materials enables the hospitalized child to undergo an enjoyable sensory experience and even to observe its final product (Siegel et al., 2016).

Many hospitalized children do not share their distress with their families so as not to increase the anxiety of family members, but they succeed in communicating to them more easily through artistic expression (Siegel et al., 2016). Art therapy can help hospitalized children accept their new reality, “the present illness,” and work through the trauma caused by hospitalization by focusing on the emotional aspects of returning to a state of good health, rather than the acute condition that was the reason for hospitalization (Weinfeld, 2013). Following the above literature, exploring a short-term mode of art therapy for hospitalized children is important and may serve as an initial step toward developing a clear working model in this domain.

## Short-Term Dynamic Therapy

The idea of short-term dynamic treatments started to develop in the late 1980s (Barber et al., 2012). Since then, the results of many studies have supported the effectiveness of emotional short-term therapy both for individual and group work (Selva et al., 2018).

Unlike traditional therapy, short-term therapy is a targeted therapy using time limits to enable an effective and thorough therapeutic event (Perkins, 2006). Such treatment is based on psychoanalytic principles to define and understand the dynamics and distress that patients bring to the therapy session and includes cognitive and behavioral techniques that deal with symptoms and habits that inhibit healthy functioning (Gatta et al., 2019). According to Bloom (2001), in short-term treatment, the therapist plans each session as an entire episode whose aim is professional help that will lead to improvement. The therapist takes a more active part in each encounter, focusing on the goal, defining interventions, and striving to reach conclusions. This process encourages patients to continue coping with the problem even after the treatment session is over. Also, both patient and therapist relate to each encounter, whether the first or the last, as a standalone therapy session (Wilson, 2021).

For short-term treatment to be effective, the therapist must be present and active, as well as act quickly to involve the patient in the therapeutic process while creating trust, cooperation, and honest communication (Driessen et al., 2015). A study of the effectiveness of short-term treatment to improve the hospitalization experience of patients with cancer found that intervention with short-term treatment improved the patients’ mood and quality of life (Raybin & Krajicek, 2020). Several studies have reported that at times short-term treatment has a greater effect than long-term treatment on the general feeling of cancer patients (Nainis, 2008). However, the efficacy of short-term art therapy with hospitalized children has been only briefly studied and discussed (Councill, 2012).

## The Current Study

Given that art therapy is an effective method of emotional intervention for the coping of hospitalized children and following the adjustment of this practice to a short-term mode, the aim of this study was to examine the perceptions, experiences, and attitudes of art therapists who conduct short-term art therapy in hospitals. Our main emphasis was on reaching a better understanding of how therapists cultivate and adapt traditional art therapy to the conditions of hospitalization and its short duration. We hope this understanding will contribute to the development of a clear short-term art therapy working model for hospitalized children.

## Methods

The study used a qualitative research design, which enables the researcher to understand the perceptions, attitudes, and experiences of the study participants in depth through semi-structured qualitative interviews, as well as to listen to the unique and subjective voice of each interviewee (Shkedi, 2003). This type of research allows us to investigate areas where the professional literature to date offers little information (Hill et al., 2005).

### Participants

This study was based on semi-structured interviews conducted with 10 art therapists who worked in three educational centers in various hospitals throughout Israel. All the caregivers were women (e.g., 99% of art therapists in that work with children are females) (Cohen-Yatziv & Regev, 2019), aged between 34 and 62 years old, and their seniority in hospitals ranged from 3 to 20 years. All participants were certified art therapists and held a master’s degree. Six therapists worked in the department of pediatrics in a general hospital, two therapists worked in the department of pediatrics in a rehabilitation hospital, and two therapists worked in a pediatric hospital. Eight of the interviewees also worked as art therapists in the community or in private clinics.

Participants were recruited using convenience sampling and snowball sampling methods. Interviewees were selected to give expression to a variety of voices, work methods, professional experience, and experience with different populations, and to obtain several perspectives on the studied phenomenon.

### Tools

An interview guide was specifically constructed for the study, and semi-structured interviews were used. At the basis of the interview was the need to understand the art therapist’s individual experience from practicing short-term art therapy with hospitalized children and the personal meaning she attributed to this experience. The advantage of this tool lies in its ability to move from structured questions that ensure uniformity, on the one hand, to open questions that give a personal character to the conversation and provide the interviewee with freedom of expression that helps us understand their perception of the phenomenon (Shkedi, 2003).

The interview consisted of four parts. The first part focused on general demographic details such as education, scope of employment, working conditions, seniority, patient population (e.g., How and when did you become a hospital teacher? In what department/s do you work? What is your formal training? Do you practice short-term art therapy daily?). In the second part of the interview, the interviewees were asked about the characteristics of the therapeutic setting in their hospital, including patient characteristics, treatment room, therapeutic utensils, treatment time, the physical working conditions, contact with multi-disciplinary staff, contact with parents (e.g., Tell me about your practice conditions in the hospital. Tell me about the hospitalized children you work with, their parents, cooperation with the medical staff.). The third part of the interview involved questions about the components of short-term art therapy within the hospital such as goals, program, working model, and therapeutic process (e.g., How do you set the goals for therapy? Do you follow a therapeutic working model? Can you characterize the therapeutic process?). Finally, the last part of the interview queried therapists about their personal experiences from doing short-term art therapy and how they cope with these experiences (e.g., How is it for you to practice short-term art therapy with hospitalized children?).

As is customary in this kind of research, the interview guide served only as an initial framework for interviews, during the interviews, additional questions were asked to deepen the understanding of the described phenomena.

### Process

The interviewees were located either by a collective e-mail distribution to art therapists or by the main researcher, who works as an academic advisor at educational centers in several hospitals. In addition, snowball sampling was utilized, in which an interviewee referred another potential interviewee to us. After a telephone call and explanation of the nature of the study, if the potential respondent expressed consent to participate in the study, a meeting was held, usually in their home or workplace, according to the interviewee’s preference. The interviewees signed a consent form to participate in the research; the form also ensured that their privacy and anonymity would be preserved.

The length of each semi-structured interview ranged between 1 and 1½ hours. Most of the interviews were conducted in Hebrew, the mother tongue of most of the therapists and the interviewer; one interview was conducted in Arabic, the mother tongue of the therapist and the interviewer. All the interviews were recorded and transcribed. The study was approved by the ethics committee of the department of social sciences and humanities of the Tel-Hai university in Israel #12/2018-9.

### Data-Processing Method

For the initial analysis of categories and themes, we used the Narralizer qualitative analysis software 1.30 in Hebrew (https://narralizer.software.informer.com/) (2004, updated 2020). Further, and according to the CQR (consensus-based qualitative research) method, the interviews were analyzed by three judges separately, with the aim of adopting several perspectives on the grouping of the main topics arising from the interviews (Hill et al., 2005). The female judges were the part of the research team (two master’s level psychology students and a senior art therapist). Each judge analyzed each interview separately and defined domains and core ideas.

In the second stage, the three judges met and conducted a cross-analysis, in which they attempted to characterize domains and core ideas that could describe the interviews to reach a consensus. The core areas and ideas formulated during this stage were submitted to the main researcher, who then examined the data to identify possible biases in the group thinking process. Next, all the formulated ideas were grouped into themes and subthemes, as shown in Table 1. Finally, all the interviews were reexamined for comprehensive scrutiny of the analysis.

**Table 1:** Themes and Subthemes.


#	THEMES	SUBTHEMES

1	The short-term art therapy setting	Physical setting

		Time setting

		Materials

		Parental presence

		Working with a multidisciplinary team

2	The components of short-term art therapy	Therapy goals

		Therapy plan

		Patient characteristics

		The therapy process

		Therapeutic discourse


According to Hill and colleagues (2005), if a category or a theme appears in the discourse of more than half of the participants (in our case more than 6 participants), it should be termed “typical” and identified as **most** participants having so responded in a certain manner. Themes cited by fewer than half the participants, but more than 25% of participants, were termed as “some,” identified as having been responded to by some participants in a certain manner. Low-frequency topics (1-2) were associated with similar themes (Hill et al., 2005), and sometimes, if important information was added, the findings were presented in the results section.

## Results

In this section, we review the findings of the study, divided into two main areas: the **setting** of the **short-term art therapy** in the hospital and the **short-term art therapy components**, both from the point of view of the art therapists. All the interviewees cooperated and were candid during the interviews; they spoke at length and gave personal examples of their work with hospitalized children.

### The Short-Term Art Therapy Setting

#### Physical Setting

Most of the art therapists noted that the art therapy clinic is pleasant, sterile, and equipped with a variety of creative materials, but many children are unable to access the clinic because they are in isolation or have a physical limitation, and thus treatment often takes place in their room or at their bedside. This often creates a problem with privacy; besides the treatment process is interrupted by other children, parents, or medical staff that enter the room.

*“Many children are in isolation and therefore cannot reach the room, so I come to them, and this harms the privacy of the treatment.”* Interviewee 4

*“There is always the possibility of entering into the emotional treatment space that is extremely breached, in contrast to the situation in my private clinic in the community.”* Interviewee 5

#### Time Setting

Most art therapists noted that in the educational services in the hospital setting it is difficult to maintain continuity of the one session and of the therapeutic process. In principle, each treatment session lasts about 45 minutes, but in practice sessions are often interrupted several times due to medical routines. In addition, children come for an unknown period, and a typical treatment process ranges between 1-2 sessions.

*“Treatment in the community usually is an hour long and has a beginning and an end. Here treatment begins, and then the patient is called out to do a blood test or the doctor enters, so the session is interrupted.”* Interviewee 3

The lack of continuity makes it difficult to keep the focus of treatment, to work through the pain, and sometimes allows only a brief acquaintance.

*“We can’t always complete the session, and that really harms the treatment.”* Interviewee 5

*“The child starts a painting and then when we reach the stage when they explain to me what is going on in the painting, they are suddenly called to be examined or someone enters the room, and then it is very difficult to return the child’s mind to the painting.”* Interviewee 8

*“Usually, I know that I will have a single session with this child, so I try to be focused and move the treatment process more rapidly than in long-term processes.”* Interviewee 1

#### Materials

Most of the art therapists described a variety of creative materials in the treatment room, more specifically hospital materials that may provide a relevant interpretation of the patient’s experience.

*“We work with test tubes and syringes and many times with plaster bandages.”* Interviewee 1

*“I have a crate in which I store as many sterile materials as I can for the patient and for work at the bedside.”* Interviewee 4

*“We need to find materials that will not require much effort from the child because they cannot physically exert himself.”* Interviewee 5

#### Parental Presence

In the opinion of all the art therapists, contact with parents is significant and inseparable from the therapeutic process, although parents do not always cooperate.

*“The in-hospital treatment is neither individual nor dyadic … it is a kind of both … you can plainly see the relationship of the parent with the child … but you also have to remember that this is a crisis situation.”* Interviewee 10

The respondents noted that there are parents from certain population sectors who consider any sort of emotional therapy to be a source of stigma and, therefore, oppose the treatment. For those who do agree to the treatment, the parent-therapist relationship can be quite complex.

*“Sometimes the parent needs my support more than the child, and it is really impossible to get past him and to work with the child.”* Interviewee 6

Most therapists reported that they advise parents about their child’s needs in the hospital. They also noted that in contrast to therapy conducted in the community, the parent is present and may be very active in therapy. Conversely, parents are often stressed and are interested in talking about their child’s difficulty; at times they make it difficult to establish a therapeutic relationship with the child.

*“Sometimes parents need our mediation on the needs of the child; for example, you cannot talk loudly on the phone about your child’s illness, you cannot cry in front of the child and worry him, you are the adult, and the child is counting on you.”* Interviewee 3

The presence of a parent influences and changes the therapeutic process, and it is important to take this into consideration.

#### Working With a Multidisciplinary Team

Most of the art therapists mentioned that the child is in the hospital to receive medical treatment and that their work with them is not the essence of the meeting but rather an attempt to alleviate the crisis caused by hospitalization or the illness.

*“At first it was really hard to get used to the fact that you are actually not the primary player in the child’s therapy, but a sort of secondary figure.”* Interviewee 3

The medical staff are naturally in a position to control what happens to the child, and the art therapists felt that they needed to “maneuver between the raindrops” and adapt themselves to the needs of the child, the parents, and the medical system.

*“You have to work with a team that may have no idea about emotional therapy and thinks that it is unnecessary or redundant or sees it as a game or a joke.”* Interviewee 7

*“The most annoying thing is when a doctor or a nurse interrupt in the middle of the session just because this is the time that is most convenient for them, even if we are in the middle of a process or a painful moment.”* Interviewee 10

*“It’s very important that everyone who works at the hospital knows what it [art therapy] is, so that they know to refer children who need this kind of support. I feel that this is an area that not everyone truly understands, not even everyone who works at the hospital knows what art therapy is.”* Interviewee 2

*“Perhaps we can do this through workshops that explain short-term art therapy and what happens behind that closed door.”* Interviewee 6

However, most of the therapists also maintained that the hospital’s professional staff cooperated and contributed to the process, thus promoting it. The multidisciplinary staff’s cooperation and their consultations with the art therapists provide additional professional information necessary for the sake of the treatment. Most of the interviewees agreed that understanding the importance of art therapy in the hospitals is gradually improving.

*“Today, there is more collaboration and information sharing between us and the psychologists, doctors, and social workers, and even more praise for our work.”* Interviewee 4

### The Components of Short-Term Art Therapy

#### Therapy Goals

Most of the respondents agreed that short-term art therapy in the hospital has several goals, but the main goal is to increase the patient’s personal well-being by empowering them and providing space for processing immediate emotions. Short-term art therapy circumvents the need for speech and allows the child to express their experience in the hospital through creativity.

*“… to develop the ability to express emotion, to distinguish between emotions, to understand what anger is … to know how to say what they feel, and, of course, to express it through materials.”* Interviewee 2

*“Art and creativity are the child’s natural language and part of their development, so it bypasses the need for speech and, furthermore, creating is certainly another way of expressing, working in art is also ‘doing’.”* Interviewee 3.

Another purpose of the treatment is to deal with the disease and to discover the patient’s healthy aspects:

*“What distinguishes art therapy as a treatment is that the child discovers the part of him that is healthy – I am sick, but I am still functioning, I still have dreams and fantasies.”* Interviewee 4

#### Therapy Plan

Most therapists agreed that they have no structured treatment plan and no one treatment method; rather, the treatment plan is more flexible and attempts to answers the current needs of the hospitalized child:

*“Working with what is necessary right now ‘in the field,’ not what has been structured.”* Interviewee 1

*“Short-term art therapy in the hospital can be an emergency treatment, not one that delves into the patient’s past and his history, but rather, one that deals with the present hospitalization.”* Interviewee 5

Only one therapist reported that she worked with a structured program she has developed for short-term treatment:

*“I have built a model that constructs these sessions to make the most of them. The model includes three parts: observing the child, identifying the main and present need, and focusing the treatment accordingly.”* Interviewee 3

#### Patient Characteristics

Most therapists described that they worked with a heterogeneous population. Children’s ages range from 3 to 21 years, with most of them being of primary school age. Most of the children are Arabs or Jews, many are religious, they come from the village, the city, and the kibbutz, from different cultures and languages, and are hospitalized for a wide range of medical conditions. The therapists ascertained that most of the hospitalized children who come to them for treatment are in a kind of crisis, confusion, and sometimes show severe distress unrelated to their family or daily routine (which is usually the topic for art therapy in the community), but to the hospitalization itself.

*“Suddenly a disease is found that threatens them, at first they undergo a crisis period and then slowly adapt.”* Interviewee 4

*“This is a situation in which they have lost their normal daily life, the routine is completely broken.”* Interviewee 2

*“Not every child succeeds in immediately expressing and talking about his feelings in a clear way … What I sense and notice is that every child succeeds in expressing his feelings more through art.”* Interviewee 6

*“Sometimes there is no Arabic-speaking therapist in the department, and although I do not have a common language with the child or his parents, art helps me bridge the language gap.”* Interviewee 1

#### Therapy Process

According to most of the therapists interviewed, the therapeutic process in the hospital addresses the child’s current needs, almost always without delving into the child’s history. This focus is what differentiates art therapy for a hospitalized child from art therapy in the community, where the longer process allows a more comprehensive exploration of the child’s life in school and at home. The hospitalized child’s therapy focuses on the here and now. Participants noted that in the therapeutic process there is an emphasis on the child regaining a sense of control, which reflects the experience of helplessness and lack of control in hospitalization. The therapeutic process allows the child to choose, decide, and express, and to receive support in return. Their creativity becomes a place where the child is the initiator and the decision-maker, and this gives them a sense of their own capability.

*“An active state of creativity gives a sense of capability and a sense of control because it is impossible to control the disease, the tests, and the duration of the hospitalization, but when the child does something artistic and feels that he is in control, this provides a boost.”* Interviewee 3

*“To concentrate only on the experience of hospitalization and the patient’s needs ‘here and now,’ without going into the child’s background …”* Interviewee 4

#### Therapeutic Discourse

Most therapists explained that therapy with a hospitalized child requires them to be very focused on the hospitalization experience.

*“In the therapeutic discourse, the child feels that he has expanded his understanding of himself, his ability, and his illness.”* Interviewee 3

*“… to be in a place where it is possible to talk about things that are not discussed elsewhere, that they have room to vent anger and frustration. Art allows them to do this.”* Interviewee 2

They usually ask about the child’s medical condition and why are they hospitalized, who is accompanying them to the hospital and, most important, how they feel. Most children cooperate, but do not always want or know how to talk about their illness. Sometimes this is an opportunity for the art therapist to help the child turn to their parents and ask questions that, until now, have been unasked. In the Arab and Jewish ultra-Orthodox sectors, parents usually prefer not to talk about the child’s illness, so the creative materials constitute a significant place for expression of emotions. The art therapists recounted that they encourage the child and even the parents to create, and that this is sometimes accompanied by discourse, or just expressing oneself via the material can offer relief.

*“You do not really have to talk about things … often it is enough to see the colors that the child chooses and the way he works with the material, in order to share his experience and his pain.”* Interviewee 10

*“It’s very important that everyone who works at the hospital knows what it is, so that they know to refer children who need this kind of support. I feel that this is an area that not everyone truly understands, not even everyone who works at the hospital knows what art therapy is.”* Interviewee 2

*“Perhaps we can do this through workshops that explain short-term art therapy and what happens behind that closed door.”* Interviewee 6

### Summary of Findings

Most therapists emphasized that short-term art therapy is an effective tool for hospitalized children, and sometimes even for their parents. They described the unique short-term characteristics of this approach as compared to traditional art therapy in the community, by explaining the need to be focused on hospitalization. Further, they emphasized the power of art to circumvent the need for speech and expression to relieve the pain and to discover the healthy aspects of the hospitalized child. The therapists discussed the focus on the here and now, both for the child – the current feelings that have arisen from hospitalization such as fear, lack of control, pain, and for the art therapist – the brief or sometimes unknown duration of the treatment, the diffused therapeutic space, the work with the multidisciplinary staff, and parents’ presence in therapy. They argued that the therapist must make many adjustments to the traditional art therapy mode in order for short-term art therapy to be effective. Finally, some respondents contended that art therapy is still not adequately recognized in hospitals as an effective short-term treatment mode and, therefore, is not sufficiently appreciated among hospital staff.

## Discussion

The purpose of this study was to examine and better understand the unique aspects of short-term art therapy with hospitalized children. While art therapy is a well-established practice, it is mostly conducted in a long-term mode (Holmqvist et al., 2017; Metzl et al., 2016). However, many children are admitted to the hospital for relatively short stays and still need the emotional support that can be delivered by art therapists (Hen, 2020). Therefore, the present study aimed to contribute to the small body of knowledge concerning short-term art therapy (Luzzatto & Gabriel, 2011) with hospitalized children and to the future establishment of this practice. Specifically, we studied 10 art therapists who worked in educational centers in hospitals, where most of their work is short term.

Consistent with the results of previous studies that found (long-term) art therapy with hospitalized children with cancer, chronic diseases, or mental illness to be very effective (Lyshak-Stelzer et al., 2007; Nainis, 2008), our findings indicated the importance and contribution of art therapy to the healing process of hospitalized children (Metzl et al., 2016; Siegel et al., 2016). Our findings also emphasized the advantage of using art materials familiar to most children from their daily lives and the opportunity for self-expression taken from the world of children (Metzl et al., 2016). The importance of a familiar and safe space for expressing negative emotions of hospitalized children and the efficacy of the treatment in reducing tension and anxiety caused by medical conditions were highlighted as well (Eaton et al., 2007). The few studies that have examined short-term therapy in hospitalized children also indicated its efficacy in relieving emotional distress resulting from hospitalization (Barber et al., 2012; Luzzatto & Gabriel, 2011). However, the focus in the current study was more on examining the characteristics of this unique practice in hospitals, and not only on its benefits, in an attempt to contribute to developing a short-term art therapy working model.

Following this goal, our findings indicated the therapists’ increased need to be focused on the “here and now,” the immediacy, flexibility, and active mode that often characterize short-term modes of therapy (Haugvik & Mossige, 2017). Specifically, the child is being treated due to hospitalization and not because of emotional difficulties occurring in their daily routine, the unpredictable nature of the therapeutic process, and the presence of parents in the session. Our findings also pointed to the unpredictable therapeutic space due to both the child’s condition and the medical routines, and the possibility that the therapy process will include only one single session. These conditions have a significant impact on the process and the therapeutic discourse, and create a different work model that is very different from the traditional model, and one that has not yet been studied or formulated.

Despite the importance of these findings, it is important to remember that the present study is a preliminary study in three hospitals in one country, utilizing a qualitative study method whose very nature limits the generalizing of its findings. Moreover, the study was based on a convenience sampling and “snowball sampling,” which also limits generalization of the findings and may be biased by social desirability on the part of the participants.

## Conclusions

The main contribution of this study lies in its (a) examination of a phenomenon that has been scarcely studied in Israel; and (b) support for the development of an art therapy model for hospitalized children based on short-term treatment approaches. As illustrated, integrating the advantages of art therapy for hospitalized children and the benefits of short-term treatment can result in an excellent therapeutic framework for encouraging medical synergies and recovery in short-term pediatric hospitalization. We hope future studies will extend these findings by studying the experiences of children, parents, and medical professionals in relation to short-term art therapy practices in order to triangulate the findings to show where this form of therapy sits within the child’s care when hospitalised.
